# Cellular Entry of *Clostridium perfringens* Iota-Toxin and *Clostridium botulinum* C2 Toxin

**DOI:** 10.3390/toxins9080247

**Published:** 2017-08-11

**Authors:** Masaya Takehara, Teruhisa Takagishi, Soshi Seike, Masataka Oda, Yoshihiko Sakaguchi, Junzo Hisatsune, Sadayuki Ochi, Keiko Kobayashi, Masahiro Nagahama

**Affiliations:** 1 Department of Microbiology, Faculty of Pharmaceutical Sciences, Tokushima Bunri University, Yamashiro-cho, Tokushima 770-8514, Japan; mtakehara@ph.bunri-u.ac.jp (M.T.); t.takagishi@ph.bunri-u.ac.jp (T.T.); kobakei@ph.bunri-u.ac.jp (K.K.); 2 Laboratory of Molecular Microbiological Science, Faculty of Pharmaceutical Sciences, Hiroshima International University, Kure, Hiroshima 737-0112, Japan; s-seike@ps.hirokoku-u.ac.jp; 3 Department of Microbiology and Infection Control Science, Kyoto Pharmaceutical University, Yamashina, Kyoto 607-8414, Japan; moda@mb.kyoto-phu.ac.jp; 4 Department of Microbiology, Kitasato University School of Medicine, 1-15-1 Kitasato, Minami-ku, Sagamihara, Kanagawa 252-0374, Japan; ysakaguchi@med.kitasato-u.ac.jp; 5 Department of Bacteriology, Graduate school of Biomedical and Health Sciences, Hiroshima University, 1-2-3 Kasumi, Minami-ku, Hiroshima 734-8551, Japan; hisatune@hiroshima-u.ac.jp; 6 Faculty of Pharmacy, Yokohama University of Pharmacy, 601 Matano-cho, Totsuka-ku, Yokohama-shi, Kanagawa 245-0066, Japan; sadayuki.ochi@hamayaku.ac.jp

**Keywords:** clostridial binary toxin, iota-toxin, C2 toxin, cellular internalization

## Abstract

*Clostridium perfringens* iota-toxin and *Clostridium botulinum* C2 toxin are composed of two non-linked proteins, one being the enzymatic component and the other being the binding/translocation component. These latter components recognize specific receptors and oligomerize in plasma membrane lipid-rafts, mediating the uptake of the enzymatic component into the cytosol. Enzymatic components induce actin cytoskeleton disorganization through the ADP-ribosylation of actin and are responsible for cell rounding and death. This review focuses upon the recent advances in cellular internalization of clostridial binary toxins.

## 1. Introduction

Clostridial binary toxins are ADP-ribosylating toxins that utilize globular actin as a substrate and depolymerize filamentous actin capped by ADP-ribosylated actin in sensitive cells. Clostridial binary toxins are produced by a few clostridia and are categorized into two groups [1,2,3]. One group consists of *Clostridium (C.) perfringens* (type E) iota-toxin [4], *C. difficile* transferase (CDT) [5], and *C. spiroforme* iota-like toxin [6]. The other group includes C2 toxin produced by *C. botulinum* types C and D [7]. The amino acid sequences of the former three binding components are more similar to each other than to C2II. These clostridial binary toxins consist of two separate protein components: the enzymatic A component and the binding/translocation B component. The binding components Ib and C2II (B components of iota-toxin and C2 toxin, respectively) specifically recognize different cellular receptors and are implicated in the uptake of A components into the intracellular space [3,8,9,10,11]. The A component (Ia) of iota-toxin mono-ADP-ribosylates non-muscle and muscle G-actin at arginine-177. On the other hand, the A component (C2I) of C2 toxin mono-ADP-ribosylates non-muscle G-actin [12]. These binary toxins cause the depolymerization of actin filaments and sensitive cells round-up as a result. Ib internalizes the A components from either iota-like toxin or CDT, but not C2II [10,11,13]. Recent advances in our understanding of the cellular uptake of iota-toxin and C2 toxin provide fascinating insights into the mechanism of cytotoxicity.

## 2. *C. perfringens* Iota-Toxin

Iota-toxin produced by *C. perfringens* type E consists of two components, an enzymatic component (Ia) and a binding component (Ib) [2,3,10,11]. Each individual component is deficient in toxic activity, but the combination of Ia and Ib causes lethal, dermonecrotic, and cytotoxic activities. Type E strain infection leads to antibiotic-associated enterotoxemia in rabbits [14,15]. Moreover, type E strains have been associated with hemorrhagic enterocolitis and sudden death in calves and lambs [14,15]. Iota-toxin is considered to be a key virulence factor of intestinal pathogenesis.

### 2.1. Structure of Ia and Ib

Crystal structure analysis of Ia indicated that it is separated into two different domains: an N-domain, which plays a role in binding with Ib, and a C-domain, which is responsible for NAD binding and ADP-ribosylating activity [16]. Previously, we reported the crystal structure of a complex consisting of Ia, actin monomer, and a hydrolysis-resistant NAD^+^ derivative [17]. On the basis of the structure of this complex, Tyr-62 and Arg-248 in Ia were shown to be critical for the Ia/actin interaction. A few conformational “snapshots” were identified, indicating that the formation of the Ia/actin complex formation occurs as part of the ADP-ribosyltransferase-catalyzed reaction. In addition, critical catalytic residues of Ia and of actin were identified. The structures confirmed a “strain-alleviation model” of ADP-ribosylation [18]. This finding suggested that all ADP-ribosyltransferases, including mono- and poly-ADP-ribosyltransferases, share a common catalytic mechanism.

Ib is produced as an inactive form (100 kDa). The active form of Ib (80 kDa) is generated by the proteolytic removal of a 20 kDa N-terminal fragment from the inactive form [1]. Ib shares 39% similarity overall with C2II (binding component of C2 toxin) [19]. Ib contains four distinct domains [2,3,10,11,19]. Domain 1 (N-terminal domain) provides the binding site for Ia; domain 4 (C-terminal domain) is a potential binding site for the host cell receptor; and domains 2 and 3 are respectively involved in oligomer assembly and pore formation. Ib assembles into heptamers, in a ring-shaped structure, which play a role in the translocation of Ia into the cytoplasm after internalization [10,11,19]. 

### 2.2. Binding and Internalization of Iota-Toxin

It has been reported that lipolysis-stimulated lipoprotein receptor (LSR) is a host cellular receptor for Ib [20,21], with iota-toxin entering host cells via an LSR-mediated process. Recently, we reported that domain 4 of Ib (Ib442-664) binds to LSR in a tricellular tight junction (tTJ) [22]. This binding led to the removal of LSR from the tTJ, which enhanced the permeation of macromolecular solutes, indicating that Ib442-664 is a modification factor of the tTJ barrier. This confirmed that domain 4 of Ib works as a receptor recognition site [2,3,10,19]. In contrast, it has also been shown that iota-toxin enters the host cells via cell-surface antigen CD44-dependent endocytosis [23].

Lipid raft membrane microdomains have been shown to serve as cell surface platforms for clustering bacteria, viruses, and several toxins [24,25,26]. These ligands have been reported to invade host cells through binding to lipid rafts. In addition, Ib oligomer clustered in plasma membrane lipid rafts as well as Ia associated with Ib oligomer invade the host cells [9,27]. Ib monomer is observed in lipid rafts and non-lipid raft fractions in whole plasma membrane at 37 °C, revealing that the Ib receptor is distributed throughout the plasma membranes. Thus, the receptor is not constrained to membrane lipid rafts. Because Ib oligomer is observed in membrane lipid rafts after treatment at 37 °C, the binding of Ib to the receptor causes movement from non-lipid rafts to lipid rafts, resulting in the formation of oligomers in the microdomains. Ib421-664 inhibited Ib binding to the target cells [9,28], and was localized in membrane lipid rafts. On the basis of these findings, because domain 4 of Ib associates with the receptor that is distributed throughout the whole cytoplasmic membrane, the receptor, which is bound to Ib, moves into membrane lipid rafts [9]. Papatherodorou et al. [20] reported that the binding component causes the accumulation of LSR in membrane lipid rafts, and LSR accumulation is a potent trigger for the oligomerization of Ib. Moreover, CD44 is mainly observed in membrane lipid rafts obtained from host cells incubated with iota-toxin [29], and CD44 promotes the accumulation of LSR into membrane lipid rafts [30]. 

### 2.3. Intracellular Trafficking of Iota-Toxin

**Figure 1:**
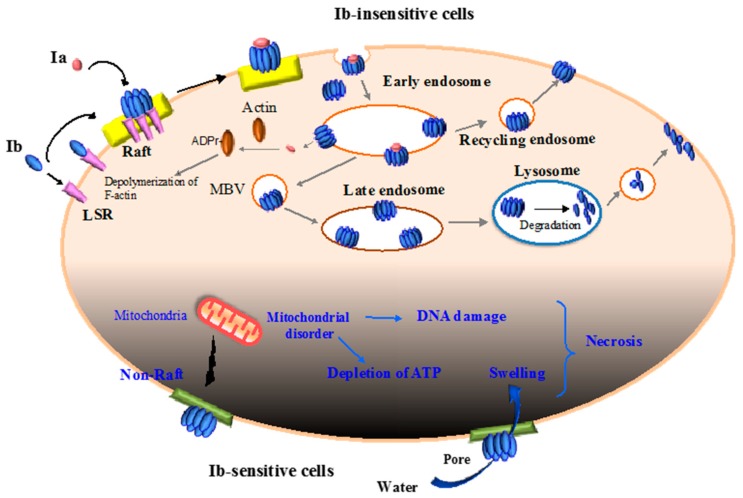
Mode of action of iota-toxin in various cells. Upper part (Ib-insensitive cells): Ib associates with a receptor (LSR: lipolysis-stimulated lipoprotein receptor) on the plasma membrane and migrates to membrane lipid raft; Ia bound to Ib oligomers forms on the rafts. Then, the Ia and Ib complex enters the cell. The complex is trafficked to the early endosome, where acidification facilitates the cytosolic release of Ia. Ia ADP-ribosylates G-actin in the cytoplasm, ultimately causing cytotoxicity. Ib is sorted into recycling endosomes and late endosomes. From recycling endosomes, Ib is sent back to the plasma membranes, and this recycling process is critical for Ib to enhance the entry of Ia. From late endosomes, Ib is delivered to lysosomes for degradation, and degraded Ib is exposed on the cell surface. Lower part (Ib-sensitive cells): Ib oligomerizes mainly in non-lipid rafts in the plasma membranes and is not internalized. Ib induces mitochondrial damage, and subsequently gives rise to the depletion of ATP and DNA damage. On the other hand, Ib causes the swelling of cells mediated by Ib pores. Finally, Ib induces cell necrosis.

*C. perfringens* iota-toxin internalizes in sensitive cells and causes cytotoxicity by utilizing the endocytic pathway [2,3,10,11,19]. Ib interacts directly with a single cellular receptor (e.g., LSR), promotes oligomerization on membrane lipid rafts, and associates with Ia [9,13,20,27]. After internalization via a Rho-independent and clathrin-dependent pathway, the toxin moves through the pathway until it reaches endocytic vesicles [13,31]. Following a 15-min incubation of Ib with host cells at 37 °C, it is detected in early endosomes (EEs) [32]. However, after 30 min, Ib is not observed in EEs and, 15–30 min later, low levels of Ib are transported to Rab11-positive recycling endosomes (REs). Therefore, a small proportion of Ib is driven back to the plasma membranes by the salvage mechanism for intracellular recycling via REs. This recycling process is critical for Ib to augment the uptake of Ia. After 30–60 min, Ib is delivered to late endosomes (LEs) and lysosomes. On the basis of these findings, Ib is internalized and delivered from EEs to REs, or LEs and lysosomes. Lysosomes migrate to cell membranes through a Ca^2+^-mediated mechanism and fuse with cell membranes [32]. Because Ib causes the elevation of intracellular Ca^2+^ from the extracellular medium, Ib induces fusion between lysosomes and cell membranes. This fusion promotes the repair of damaged membranes during Ib pore formation (Figure 1). 

### 2.4. Translocation of Ia across the Endosomal Membrane

After the endocytosis of iota-toxin, Ia passes through the endosomal membrane into the cytoplasm. This process uses the endosomal membrane-spanning pore formed by Ib. Acidic environments are essential for the translocation of Ia from EEs into the cytoplasm. Acidic pH causes structural alterations in the Ib oligomer, accelerating the insertion of the Ib oligomer into endosomal membranes and in turn the migration of Ia via oligomeric Ib pores into the cytoplasm after the pH gradient [31]. Ia is partly unfolded in order to migrate via the narrow oligomeric Ib pore into the cytoplasm. The pH-dependent transmembrane transport or cytoplasmic refolding of Ia is promoted by cytoplasmic factors containing the molecular chaperone heat shock protein 90 (Hsp90) and peptidyl-prolyl *cis/trans*-isomerase (PPIase), including cyclophilin A and FK-506 binding protein [33]. PPIase is a folding helper protein. Suppression of Hsp90 and PPIase blocks the transmembrane transport of Ia into the cytoplasm, and Ia binds to Hsp90 and PPIase in dot-blot analyses. Recently, it has been reported that an Hsp70 inhibitor blocks cytotoxicity induced by iota-toxin [34]. Hsp70 assists protein transport during transit through membranes. Hsp70, Hsp90, and PPIase cooperatively configure multi-chaperone complexes critical for the protein folding, intracellular localization, and maturation of particular proteins. The unfolded Ia has been shown to undergo potent binding with Hsp70 and PPIase compared with the native form. Together, Hsp70, Hsp-90, and PPIase are important for transport of Ia through the endosomal membrane [33,34,35].

### 2.5. Cytotoxicity of Ib

Ib alone has been shown to lack toxic activity [2,3,10,11,19]. We have reported that, after the binding of Ib to the cell surface receptor on Vero cells, it forms oligomers and creates ion-permeable pores [8]. The formation of pores by Ib has been demonstrated in planar lipid bilayers [36]. Ib creates the ion-permeable channels [36], and domains 2 and 3 in Ib are critical for oligomerization and the formation of channels, respectively [36,37]. Moreover, Ib leads to a decrease in transepithelial electrical resistance (TEER) in human intestinal epithelial Caco-2 cell monolayers [37]. We demonstrated that Ib alone has cytotoxic activity, and we examined the effects of Ib alone in eight cell lines [38]. Ib rapidly caused cell swelling, depletion of ATP, and reduction in viability among human epithelial carcinoma A431 and human lung adenocarcinoma A549 cells [38]. In MDCK cells, which are not sensitive to the cytotoxic activity of Ib, the Ib oligomer formed in membrane lipid rafts is taken up by endocytosis [9,38]. However, Ib also formed oligomers in non-raft membranes in A431 cells [38]. Additionally, Ib was present on the A431 cell surface while exhibiting its toxic activity. Long-term persistence of Ib in cell surfaces was dependent on the cell type, and internalization of Ib was linked with the survival of the challenge of Ib pores (Figure 1). Therefore, the ability of a cell type to survive membrane perforation by Ib depends on its ability to internalize Ib. Our results showed that the endocytosis of Ib is needed for host cell survival, and the function of endocytosis is an innate host defense response against pore-forming proteins [38].

Because no cell line was known to be susceptible to Ib until now, the finding that Ib induces cell death in A431 and A549 cells is a new discovery, and this may help to elucidate the contribution of Ib to the virulence of type E strains.

## 3. *C. botulinum* C2 Toxin

C2 toxin produced by *C. botulinum* type C and D is composed of an enzymatic subunit (C2I) and a binding/translocation subunit (C2II). Each protein component is generated separately and they are not linked. *C. botulinum* C2 toxin occasionally causes enteric hemorrhagic and necrotizing damage in animals, especially in avian species, which die of poisoning due to *C. botulinum* [11]. Experimentally, C2 toxin induces dermonecrosis and hemorrhagic enterocolitis in mice [11].

### 3.1. Structure of C2I and C2II

From its crystal structure, C2I is composed of two nearly equal-sized domains of about 200 residues [39]. Residues 1 to 87 of the N-terminal domain serve as a binding site for C2II. The C-terminal domain of C2I is involved in actin ADP-ribosyltransferase activity [2,10,19,39]. The C-terminal domain harbors highly conserved catalytic residues amongst bacterial ADP-ribosyltransferases.

C2II has to be activated by proteolytically cleaving a ~20 kDa N-terminal fragment (C2IIa) [2,10,11]. The structure of C2II has been determined and indicates that its structure is similar to that of protective antigen (PA), the binding component of *Bacillus anthracis* [39]. C2II exhibits four functional domains similar to iota-toxin [2,10,11]. The N-terminal domain (domain 1) includes the docking region with C2I; domain 2 is essential for the oligomer formation; the biological function of domain 3 is unclear; and the C-terminal domain (domain 4) plays a role in the recognition of the host cell receptor.

### 3.2. Binding and Internalization of C2 Toxin

Domain 4 of C2II has been determined as the binding domain for the host cell receptor [2,10,11]. C2II oligomers bind to asparagine-linked carbohydrates at the cell surface receptor [40]. The low level of amino-acid identity (lower than approximately 10%) between the sequence of these binding domains agrees well with the fact that each one binds to different receptors.

**Figure 2:**
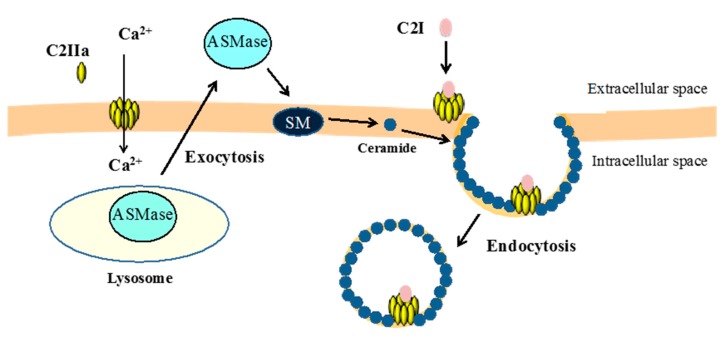
Initial step in the internalization of C2 toxin. Extracellular Ca^2+^ entry into the cytosol via a C2IIa pore. An increase in the intracellular Ca^2+^ concentration evokes lysosomal exocytosis. Lysosomal acid sphingomyelinase (ASMase) is secreted to the outer plasma membrane, where it hydrolyzes sphingomyelin into ceramide. Ceramide self-assembles into microdomains that bend to the intracellular space, elaborating endosomes that endocytose the C2I and C2IIa complex.

Recently, we demonstrated that C2 toxin needs the activity of acid sphingomyelinase (ASMase) during the initial step of endocytosis [41]. Several bacterial pore-forming cytotoxic proteins cause calcium entry and provoke the exocytosis of lysosomes, leading to the release of lysosomal ASMase to the extracellular medium [42,43]. Next, ASMase converts sphingomyelin in the outer leaflet of the plasma membrane to ceramide [44]. Then, ceramide self-assembles into ceramide-enriched microdomains that bud into cytoplasmic membranes, creating endosomes [45]. Namely, the hydrolysis of sphingomyelin by ASMase secreted from the exocytosis of lysosomes produces plasma membrane microdomains enriched in ceramide, leading to endocytosis [44,45]. C2IIa causes the entry of extracellular calcium into sensitive cells, and the cytotoxic activity of C2 toxin is increased in calcium-containing medium [41]. Blockers of lysosomal exocytosis and ASMase inhibit the cytotoxic activity of C2 toxin. Moreover, C2IIa induces the release of ASMase due to the exocytosis of lysosomes. Then, C2 toxin induces ceramide production in plasma membranes. On the basis of these findings, it is concluded that ASMase activity is required for C2 toxin entry into host cells [41]. In Figure 2, we show a hallmark of the key role of ASMase in the endocytosis of C2 toxin in sensitive cells.

C2IIa oligomer binds to plasma membrane lipid rafts [46]. By surface plasmon resonance analysis, it has been shown that C2I associates with oligomers of C2IIa but not with C2IIa monomers [46]. The binding of C2I to lipid raft-associated C2IIa oligomers induces rapid internalization of C2 toxin into host cells. The entry of C2 toxin proceeds through membrane lipid rafts, indicating that these structures include fundamental factors facilitating the internalization of C2 toxin. Hence, the C2I-C2IIa complex is endocytosed via plasma membrane lipid rafts [46].

Phosphatidylinositol 3-kinase (PI3K) and Akt inhibitors blocked the endocytosis of C2 toxin in the host cells and the cytotoxic effect of C2 toxin [46]. In fact, C2 toxin induced the activation of PI3K and the phosphorylation of Akt [46]. As mentioned above, C2 toxin causes the production of ceramide as a result of the ASMase activity [41]. Ceramide is metabolized to ceramide-1-phosphate (C1P). As stimulation of a C1P receptor is known to activate the PI3K/Akt signaling pathway, activation of this pathway by C2 toxin is needed for the production of C1P [46]. On the other hand, activation of the PI3K/Akt signaling pathway promotes cell survival [47,48]. Accordingly, antagonistic modes of action on the cytotoxicity act as the cellular defense mechanism against internalizing C2 toxin.

### 3.3. Intracellular Trafficking of C2 Toxin

The association of C2I with C2IIa oligomer on membrane lipid rafts induces PI3K-Akt signaling pathway activation and then internalization [46]. C2 toxin is trafficked to EEs, where the release of C2I into the cytosol occurs. C2I translocation is promoted by the actions of Hsp90 and PPIase, as well as translocating Ia [49,50]. In the cytoplasm, C2I catalyzes the ADP-ribosylation of G-actin, ensuring the depolymerization of filamentous actin and the rounding up of sensitive cells. C2IIa is transferred to LEs and lysosomes [51]. Conversely, a portion of C2IIa is transported to REs. C2IIa reappearing on the cell membrane could re-associate with C2I. These findings demonstrate that C2IIa is endocytosed and sorted from EEs to REs or LEs and lysosomes [51].

## 4. Conclusions

Iota-toxin and C2 toxin belong to the bacterial AB toxin family. These toxins possess the potency to internalize into cells and to release an enzymatic component into the cytoplasmic space. The amino acid sequence identity of the binding component of iota toxin and C2 toxin is rather low. This difference is reflected in differences in receptors. Iota toxin receptor is a proteinaceous receptor such as LSR receptor and CD44. C2 toxin is a sugar receptor. The invasion process of both toxins has much in common. Pores formed by oligomers of binding components promote the release of enzymatic components from EEs into the cytoplasmic space. Iota-toxin and C2 toxin may become important tools to induce the entry of efficacious substances or targeted therapeutic agents into particular cells. On the other hand, inhibitors of internalization and intracellular trafficking have the potential for use as useful therapeutic treatments for infectious diseases. 

## References

[1] Gibert M., Petit L., Raffestin S., Okabe A., Popoff M.R. (2000). *Clostridium perfringens* iota-toxin requires activation of both binding and enzymatic components for cytopathic activity. Infect. Immun..

[2] Popoff M.R., Boquet P. (2009). Clostridial toxins. Future Microbiol..

[3] Sakurai J., Nagahama M., Oda M., Tsuge H., Kobayashi K. (2009). *Clostridium perfringens* iota-toxin: Structure and function. Toxins.

[4] Stiles B.G., Wilkins T.D. (1986). Purification and characterization of *Clostridium perfringens* iota-oxin: Dependence on two nonlinked proteins for biological activity. Infect. Immun..

[5] Gülke I., Pfeifer G., Liese J., Fritz M., Hofmann F., Aktories K., Barth H. (2001). Characterization of the enzymatic component of the ADP-ribosyltransferase toxin. CDTa from *Clostridium difficile*. Infect. Immun..

[6] Popoff M.R., Boquet P. (1988). *Clostridium spiroforme* toxin is a binary toxin which ADP-ribosylates cellular actin. Biochem. Biophys. Res. Commun..

[7] Aktories K., Bärmann M., Ohishi I., Tsuyama S., Jakobs K.H., Habermann E. (1986). Botulinum C2 toxin ADP-ribosylates actin. Nature.

[8] Nagahama M., Nagayasu K., Kobayashi K., Sakurai J. (2002). Binding component of *Clostridium perfringens* iota-toxin induces endocytosis in Vero cells. Infect. Immun..

[9] Nagahama M., Yamaguchi A., Hagiyama T., Ohkubo N., Kobayashi K., Sakurai J. (2004). Binding and internalization of *Clostridium perfringens* iota-toxin in lipid rafts. Infect. Immun..

[10] Aktories K., Lang A.E., Schwan C., Mannherz H.G. (2011). Actin as target for modification by bacterial protein toxins. FEBS J..

[11] Knapp O., Benz R., Popoff M.R. (2016). Pore-forming activity of clostridial binary toxins. Biochim. Biophys. Acta.

[12] Mauss S., Chaponnier C., Just I., Aktories K., Gabbiani G. (1990). ADP-ribosylation of actin isoforms by *Clostridium botulinum* C2 toxin and *Clostridium perfringens* iota toxin. Eur. J. Biochem..

[13] Gibert M., Monier M.N., Ruez R., Hale M.L., Stiles B.G., Benmerah A., Johannes L., Lamaze C., Popoff M.R. (2011). Endocytosis and toxicity of clostridial binary toxins depend on a clathrin-independent pathway regulated by Rho-GDI. Cell. Microbiol..

[14] Songer J.G. (1996). Clostridial enteric diseases of domestic animals. Clin. Microbiol. Rev..

[15] Li J., Adams V., Bannam T.L., Miyamoto K., Garcia J.P., Uzal F.A., Rood J.I., McClane B.A. (2013). Toxin plasmids of *Clostridium perfringens*. Microbiol. Mol. Biol. Rev..

[16] Tsuge H., Nagahama M., Nishimura H., Hisatsune J., Sakaguchi Y., Itogawa Y., Katunuma N., Sakurai J. (2003). Crystal structure and site-directed mutagenesis of enzymatic components from *Clostridium perfringens* iota-toxin. J. Mol. Biol..

[17] Tsuge H., Nagahama M., Oda M., Iwamoto S., Utsunomiya H., Marquez V.E., Katunuma N., Nishizawa M., Sakurai J. (2008). Structural basis of actin recognition and arginineADP-ribosylation by *Clostridium perfringens* iota-toxin. Proc. Natl. Acad. Sci. USA.

[18] Tsurumura T., Tsumori Y., Qiu H., Oda M., Sakurai J., Nagahama M., Tsuge H. (2013). Arginine ADP-ribosylation mechanism based on structural snapshots of iota-toxin and actin complex. Proc. Natl. Acad. Sci. USA.

[19] Barth H., Aktories K., Popoff M.R., Stiles B.G. (2004). Binary bacterial toxins: Biochemistry, biology, and applications of common *Clostridium* and *Bacillus* proteins. Microbiol. Mol. Biol. Rev..

[20] Papatheodorou P., Carette J.E., Bell G.W., Schwan C., Guttenberg G., Brummelkamp T.R., Aktories K. (2011). Lipolysis-stimulated lipoprotein receptor (LSR) is the host receptor for the binary toxin *Clostridium difficile* transferase (CDT). Proc. Natl. Acad. Sci. USA.

[21] Schmidt G., Papatheodorou P., Aktories K. (2015). Novel receptors for bacterial protein toxins. Curr. Opin. Microbiol..

[22] Krug S.M., Hayaishi T., Iguchi D., Watari A., Takahashi A., Fromm M., Nagahama M., Takeda H., Okada Y., Sawasaki T. (2017). Angubindin-1, a novel paracellular absorption enhancer acting at the tricellular tight junction. J. Control. Release.

[23] Wigelsworth D.J., Ruthel G., Schnell L., Herrlich P., Blonder J., Veenstra T.D., Carman R.J., Wilkins T.D., Van Nhieu G.T., Pauillac S. (2012). CD44 promotes intoxication by the clostridial iota-family toxins. PLoS ONE.

[24] Mañes S., del Real G., Martínez-A C. (2003). Pathogens: Raft hijackers. Nat. Rev. Immunol..

[25] Zaas D.W., Duncan M., Wright J.R., Abraham S.N. (2005). The role of lipid rafts in the pathogenesis of bacterial infections. Biochim. Biophys. Acta.

[26] Riethmüller J., Riehle A., Grassmé H., Gulbins E. (2006). Membrane rafts in host-pathogen interactions. Biochim. Biophys. Acta.

[27] Hale M.L., Marvaud J.C., Popoff M.R., Stiles B.G. (2004). Detergent-resistant membrane microdomains facilitate Ib oligomer formation and biological activity of *Clostridium perfringens* iota-toxin. Infect. Immun..

[28] Marvaud J.C., Smith T., Hale M.L., Popoff M.R., Smith L.A., Stiles B.G. (2001). *Clostridium perfringens* iota-toxin: Mapping of receptor binding and Ia docking domains on Ib. Infect. Immun..

[29] Blonder J., Hale M.L., Chan K.C., Yu L.R., Lucas D.A., Conrads T.P., Zhou M., Popoff M.R., Issaq H.J., Stiles B.G. (2005). Quantitative profiling of the detergent-resistant membrane proteome of iota-b toxin induced vero cells. J. Proteome Res..

[30] Papatheodorou P., Hornuss D., Nölke T., Hemmasi S., Castonguay J., Picchianti M., Aktories K. (2013). *Clostridium difficile* binary toxin CDT induces clustering of the lipolysis-stimulated lipoprotein receptor into lipid rafts. mBio.

[31] Gibert M., Marvaud J.C., Pereira Y., Hale M.L., Stiles B.G., Boquet P., Lamaze C., Popoff M.R. (2007). Differential requirement for the translocation of clostridial binary toxins: Iota toxin requires a membrane potential gradient. FEBS Lett..

[32] Nagahama M., Umezaki M., Tashiro R., Oda M., Kobayashi K., Shibutani M., Takagishi T., Ishidoh K., Fukuda M., Sakurai J. (2012). Intracellular trafficking of *Clostridium perfringens* iota-toxin b. Infect. Immun..

[33] Kaiser E., Kroll C., Ernst K., Schwan C., Popoff M., Fischer G., Buchner J., Aktories K., Barth H. (2011). Membrane translocation of binary actin-ADP-ribosylating toxins from *Clostridium difficile* and *Clostridium perfringens* is facilitated by cyclophilin A and Hsp90. Infect. Immun..

[34] Ernst K., Liebscher M., Mathea S., Granzhan A., Schmid J., Popoff M.R., Ihmels H., Barth H., Schiene-Fischer C. (2016). A novel Hsp70 inhibitor prevents cell intoxication with the actin ADP-ribosylating *Clostridium perfringens* iota toxin. Sci. Rep..

[35] Ernst K., Schmid J., Beck M., Hägele M., Hohwieler M., Hauff P., Ückert A.K., Anastasia A., Fauler M., Jank T. (2017). Hsp70 facilitates trans-membrane transport of bacterial ADP-ribosylating toxins into the cytosol of mammalian cells. Sci. Rep..

[36] Knapp O., Benz R., Gibert M., Marvaud J.C., Popoff M.R. (2002). Interaction of *Clostridium perfringens* iota-toxin with lipid bilayer membranes. Demonstration of channel formation by the activated binding component Ib and channel block by the enzyme component Ia. J. Biol. Chem..

[37] Richard J.F., Mainguy G., Gibert M., Marvaud J.C., Stiles B.G., Popoff M.R. (2002). Transcytosis of iota-toxin across polarized CaCo-2 cells. Mol. Microbiol..

[38] Nagahama M., Umezaki M., Oda M., Kobayashi K., Tone S., Suda T., Ishidoh K., Sakurai J. (2011). *Clostridium perfringens* iota-toxin b induces rapid cell necrosis. Infect. Immun..

[39] Schleberger C., Hochmann H., Barth H., Aktories K., Schulz G.E. (2006). Structure and action of the binary C2 toxin from *Clostridium botulinum*. J. Mol. Biol..

[40] Eckhardt M., Barth H., Blöcker D., Aktories K. (2000). Binding of *Clostridium botulinum* C2 toxin to asparagine-linked complex and hybrid carbohydrates. J. Biol. Chem..

[41] Nagahama M., Takehara M., Takagishi T., Seike S., Miyamoto K., Kobayashi K. (2017). Cellular uptake of *Clostridium botulinum* C2 toxin requires acid sphingomyelinase activity. Infect. Immun..

[42] Idone V., Tam C., Andrews N.W. (2008). Two-way traffic on the road to plasma membrane repair. Trends Cell Biol..

[43] Los F.C., Randis T.M., Aroian R.V., Ratner A.J. (2013). Role of pore-forming toxins in bacterial infectious diseases. Microbiol. Mol. Biol. Rev..

[44] Tam C., Idone V., Devlin C., Fernandes M.C., Flannery A., He X., Schuchman E., Tabas I., Andrews N.W. (2010). Exocytosis of acid sphingomyelinase by wounded cells promotes endocytosis and plasma membrane repair. J. Cell Biol..

[45] Corrotte M., Fernandes M.C., Tam C., Andrews N.W. (2012). Toxin pores endocytosed during plasma membrane repair traffic into the lumen of MVBs for degradation. Traffic.

[46] Nagahama M., Hagiyama T., Kojima T., Aoyanagi K., Takahashi C., Oda M., Sakaguchi Y., Oguma K., Sakurai J. (2009). Binding and internalization of *Clostridium botulinum* C2 toxin. Infect. Immun..

[47] Gómez-Muñoz A. (2006). Ceramide 1-phosphate/ceramide, a switch between life and death. Biochim. Biophys. Acta.

[48] Rivera I.G., Ordoñez M., Presa N., Gomez-Larrauri A., Simón J., Trueba M., Gomez-Muñoz A. (2015). Sphingomyelinase D/ceramide 1-phosphate in cell survival and inflammation. Toxins.

[49] Barth H. (2011). Exploring the role of host cell chaperones/PPIases during cellular up-take of bacterial ADP-ribosylating toxins as basis for novel pharmacological strategies to protect mammalian cells against these virulence factors. Naunyn-Schmiedebergs Arch. Pharmacol..

[50] Kaiser E., Böhm N., Ernst K., Langer S., Schwan C., Aktories K., Popoff M., Fischer G., Barth H. (2012). FK506-binding protein 51 interacts with *Clostridium botulinum* C2 toxin and FK506 inhibits membrane translocation of the toxin in mammalian cells. Cell. Microbiol..

[51] Nagahama M., Takahashi C., Aoyanagi K., Tashiro R., Kobayashi K., Sakaguchi Y., Ishidoh K., Sakurai J. (2014). Intracellular trafficking of *Clostridium botulinum* C2 toxin. Toxicon.

